# Retraction: AD-Diff: enhancing Alzheimer's disease prediction accuracy through multimodal fusion

**DOI:** 10.3389/fncom.2026.1957950

**Published:** 2026-08-06

**Authors:** Frontiers Editorial Office

The journal retracts the article cited above.

Following concerns regarding the originality of the article, an investigation was conducted in accordance with Frontiers' policies. It was found that the complaint was valid and that the article should be retracted, because of an unacceptable level of similarity to “GFE-Mamba: Mamba-based AD Multi-modal Progression Assessment via Generative Feature Extraction from MCI,” posted on arXiv as arXiv:2407.15719v1.

This retraction was approved by the Chief Executive Editor of Frontiers. The author received a communication regarding the retraction and had a chance to respond. This communication is recorded by the publisher.

Frontiers would like to thank the concerned reader who contacted us regarding the published article.

